# Sustainable crop intensification through surface water irrigation in Bangladesh? A geospatial assessment of landscape-scale production potential

**DOI:** 10.1016/j.landusepol.2016.10.001

**Published:** 2017-01

**Authors:** Timothy J. Krupnik, Urs Schulthess, Zia Uddin Ahmed, Andrew J. McDonald

**Affiliations:** a International Maize and Wheat Improvement Center (CIMMYT) – Bangladesh, House 10/B, Road 53, Gulshan-2, Dhaka 1213, Bangladesh; b International Maize and Wheat Improvement Center (CIMMYT) – South Asia Regional Office, South Asia Regional Office, Botany Division, NARC, Khumaltar, Lalitpur District, Nepal

**Keywords:** Sustainable intensification, Irrigation, Land use classification, Double cropping, Technology targeting, Ecosystem services

## Abstract

Changing dietary preferences and population growth in South Asia have resulted in increasing demand for wheat and maize, along side high and sustained demand for rice. In the highly productive northwestern Indo-Gangetic Plains of South Asia, farmers utilize groundwater irrigation to assure that at least two of these crops are sequenced on the same field within the same year. Such double cropping has had a significant and positive influence on regional agricultural productivity. But in the risk-prone and food insecure lower Eastern Indo-Gangetic Plains (EIGP), cropping is less intensive. During the dryer winter months, arable land is frequently fallowed or devoted to lower yielding rainfed legumes. Seeing opportunity to boost cereals production, particularly for rice, donors and land use policy makers have consequently reprioritized agricultural development investments in this impoverished region. Tapping groundwater for irrigation and intensified double cropping, however, is unlikely to be economically viable or environmentally sound in the EIGP. Constraints include saline shallow water tables and the prohibitively high installation and energetic extraction costs from deeper freshwater aquifers. The network of largely underutilized rivers and natural canals in the EIGP could conversely be tapped to provide less energetically and economically costly surface water irrigation (SWI). This approach is now championed by the Government of Bangladesh, which has requested USD 500 million from donors to implement land and water use policies to facilitate SWI and double cropping. Precise geospatial assessment of where freshwater flows are most prominent, or where viable fallow or low production intensity cropland is most common, however remains lacking. In response, we used remotely sensed data to identify agricultural land, detect the temporal availability of freshwater in rivers and canals, and assess crop production intensity over a three-year study period in a 33,750 km^2^ case study area in southwestern Bangladesh. We combined these data with georeferenced and temporally explicitly soil and water salinity information, in addition to relative elevation classifications, in order to examine the extent of winter fallows and low productivity rainfed cropland that could be irrigated by small-scale surface water pumps. Applying observations of irrigated crop sowing dates and yields from 510 wheat, 550 maize, and 553 rice farmers, we also modeled crop intensification production scenarios within the case study area. We conservatively estimate that at least 20,800 and 103,000 ha of fallow and rainfed cropland, respectively, could be brought into intensified double cropping using SWI. Scenario analysis indicates that if 25%–75% of the fallow or low-intensity land were converted to irrigated maize, national aggregate production could increase by 10–14% or 29–42%, respectively. Conversion to wheat would conversely boost national production by 9–10% or 26–31%. Irrigated rice is however unlikely to contribute >3%. In aggregate, these actions could generate between USD 36–108 million of revenue annually among farmers. Intensification therefore has important land use policy and food and income security implications, helping to rationalizei SWI investments. Crop choice, water resource allocation, and water governance will however remain crucial considerations for irrigation planners.

## Highlights

• Arable land area in South Asia is declining while cereal demand is increasing.
• Bangladesh emphasizes surface water irrigation (SWI) for sustainable intensification.
• Remotely sensed, geospatial, and farmers’ yield data were integrated to target SWI.
• 20,800 and 103,000 ha of fallow and rainfed cropland could benefit from SWI.
• SWI policy could substantially increase maize and wheat, but not rice production.

## Graphical abstract

**Figure:**
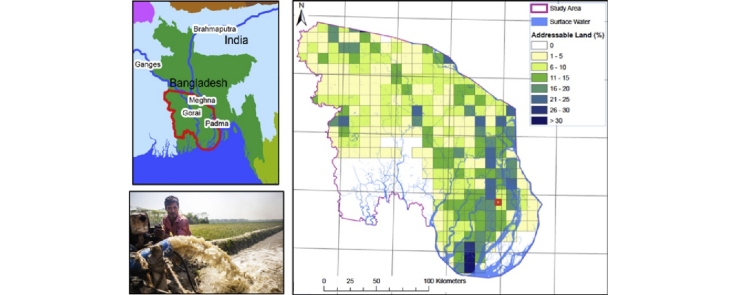

## 1 Introduction

Global food requirements are projected to increase for at least four decades before they plateau, with a doubling of staple crop production required by 2050 (Godfray et al., 2010). In South Asia, average wheat (*Triticum aestivum*), maize (*Zea* mays), and rice (*Oryza sativa*) yields have however increased by only 2.2%, 1.4%, and 1.3%, respectively, since the 1960s (FAOSTAT, 2013). Rather than raising yield, crop production can be increased by expanding cultivated land area, though conversion of natural ecosystems to agricultural land uses is at odds with sustainable development goals (UN DESA, 2016). The potential for agricultural expansion in South Asia is also limited because most arable land is already cropped for at least part of the year (FAO, 2014). Farm area per capita in Bangladesh, India, Nepal, and Pakistan, for example, has shrunk by an average of 63% since 1961, to approximately 0.1 ha person^−1^ (World Bank, 2015).

Sustainable intensification (SI) has been proposed as an alternative to agricultural area expansion (Godfray et al., 2010, Pretty and Bharucha, 2014). SI aims to augment land productivity by increasing resource use efficiency while minimizing environmental externalities (Pretty and Bharucha, 2014). Although critiqued as placing more emphasis on intensification than sustainability (Petersen and Snapp, 2015), the approach has gained considerable popularity among researchers, international donors, and policymakers. An important SI strategy entails increasing the number of crops grown per year on the same land, thereby raising yield per unit of area-time, while also minimizing land expansion and consequent biodiversity loss (Krupnik et al., 2015a, Pretty and Bharucha, 2014). Achieving such ‘double cropping’ will often require irrigation. Concentrated in the western IGP, India ranks among the globe’s most intensively irrigated countries (AQUASTAT, 2016), leading to the perception that much of the potentially irrigable land in South Asia is already double cropped (cf. de Fraiture and Wichelns, 2010, Godfray et al., 2010). Most research efforts have consequently focused on closing yield gaps under relatively favorable irrigated conditions, primarily in the western IGP. Less emphasis has conversely been placed on investigating the ways in which dry season fallowed or rainfed land can be intensified.

By moderating the risk inherent to water-limited crop production, energy-efficient irrigation could provide a solid foundation for SI (Krupnik et al., 2015b, Molden, 2007). The geographical distribution of irrigation development in the eastern IGP is however uneven. In West Bengal and coastal Bangladesh, for example, dry season land fallowing is common. Groundwater irrigation facilities are lacking, especially near the coast where shallow aquifers may be saline (Bell et al., 2015). Where environmental conditions are more permitting, the cost of deep tube well drilling and installation is still out of reach for most resource-poor farmers (Bernier et al., 2016).

SI also aims to harness ecosystem services to sustain crop productivity (Pretty and Bharucha, 2014). This can include freshwater supply and regulation for irrigation (Aylward et al., 2005, Power, 2010). While groundwater access is challenging, Southern Bangladesh is known for its dense network of rivers and natural canals that comprise part of a tidal ecosystem that conveys fresh surface water proximal to farmland. Southward freshwater flow also pushes back oceanic saltwater intrusion during the dry season. Surface water irrigation (SWI) is also considerably less energy demanding than groundwater extraction, with a lower carbon footprint (Shah, 2009). New surface water pumps in Bangladesh also show up to 51% increased fuel efficiency relative to their conventional low-lift counterparts. This can resulti in both environmental and economic benefits (Krupnik et al., 2015b). The extent of land in the eastern IGP that can be addressed with SWI has however not been quantified or made spatially explicit, providing the central motivating question for this study.

The Government of Bangladesh (GoB) recently adopted policy calling for investment of over USD 7 billion to support agricultural development in southern Bangladesh (MOA and FAO, 2013). Approximately 1.7 million farming households in this area fallow their land after the monsoon, contributing to food insecurity and subsistence below the poverty line (World Bank, 2010). An anticipated USD 500 million of the funds requested by the GoB are to be allocated for SWI initiatives to transition farmers from rice-fallow or rainfed systems into intensified double cropping. Further emphasis is placed on increasing dry season *boro* rice production in southern Bangladesh (MOA and FAO, 2013), partly to offset increasing production and energy subsidy costs in existing *boro* areas in the north reliant on groundwater irrigation. The viability of this approach has however been questioned given the southern region’s soil and water salinity constraints, problems with coordinated water governance, and concerns over the anticipated long-term effects of climate change (Bell et al., 2015, Bernier et al., 2016, Qureshi et al., 2015).

SWI can be applied in two ways: through large irrigation schemes and elaborate canal networks, or through smaller, decentralized command areas managed by independent water sellers who channel water to farmers’ fields through flexible hose piping or farmer maintained earthen canals (Krupnik et al., 2015b). The decentralized approach builds on models pervasive in Bangladesh’s groundwater irrigated areas (Palmer-Jones, 2001), and may provide a more immediate solution for southern Bangladesh. Investments to expand SWI are however complicated by the region’s crop, soil, and hydrological complexity. Upstream blockages, diversions, siltation and tidal dynamics complicate year-round water availability. Temporally dynamic water and soil salinity also pose problems in the coastal fringe, as can late-vacating monsoon season floodwaters that delay timely dry season crop establishment (Krupnik et al., 2015a). It is therefore important to geographically assess freshwater availability and target areas and crops that can benefit the most from SWI.

This paper responds to this need using a 33,750 km^2^ study area in the southwest of Bangladesh. Our objectives were to assess the extent of fallow and rainfed cropland that can be brought into intensified dry season cereal production through SWI. Satellite imagery is now freely available that enables detailed land-use suitability analyses. Additional geospatial environmental data were integrated to identify the land area upon which SWI is most feasible. We also collected data from over 1,600 farmers and modeled a number of intensified production scenarios to examine the potential contribution that SWI could make to Bangladesh’s national cereal production.

## 2 Materials and methods

### 2.1 Study area

**Fig. 1:**
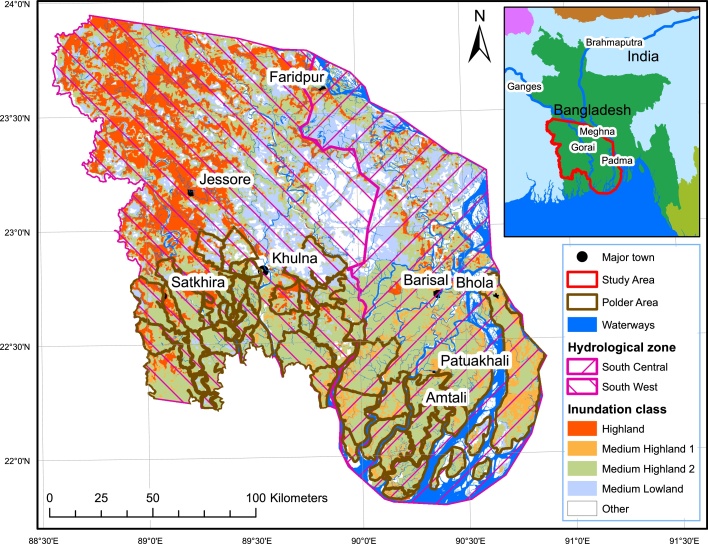
The study area in southwestern Bangladesh. For interpretation of the references to colour in this figure legend, the reader is referred to the online version of this article.

**Fig. 2:**
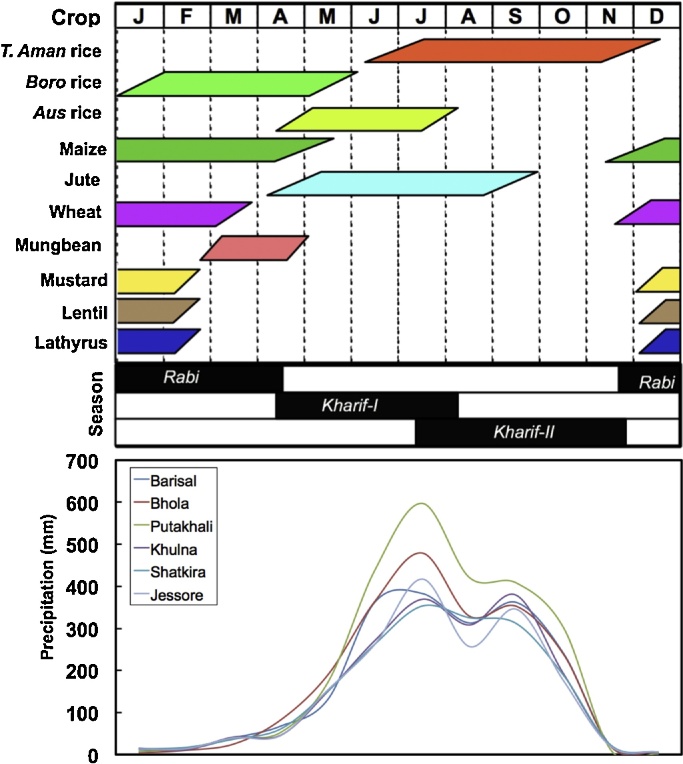
Predominant crops, seasons, and precipitation patterns (averaged from 2002 to 2012) for major districts of interest in the study area. Adapted from Brammer (2013). For interpretation of the references to colour in this figure legend, the reader is referred to the online version of this article.

We studied a 33,750 km^2^ area (24°2′23′′N–21°46′38′′S and 88°33′33′′W–90°52′44′′E) in southwestern Bangladesh, encompassing all land to the south and west of the Ganges and Padma rivers, excluding the Sundarbans forest (Fig. 1). Coastal farmers experience soil and water salinity that builds as the dry season progresses, although southward freshwater flow regulates and lowers salinity concentrations (Chowdhury, 2010, Rawson, 2011). Precipitation is unimodal (Fig. 2). The study area encompasses two major hydrological units, including the southwest and south-central zones.

Differences in monsoon season flooding depth and duration are used to define cropland suitability in Bangladesh. Classifications include Highland (not flooded during the monsoon), Medium-Highland-1 and -2 (maximum monsoon flooding between 0 and 45 and 46–90 cm, respectively), Medium-Lowland (flooding above 90–180 cm), and Lowlands (>180 cm monsoon flooding) (Brammer, 2013). Lowlands may remain waterlogged in the early dry season, complicating field accessibility. When early monsoon season rains begin, lowlands are also prone to flash and stagnant flooding. At higher relative landscape positions, flood depth classifications are generally inversely related to the time at which land can be sown to dry season crops, as these landscape positions tend to drain more quickly. Maize and wheat, for example, require earlier crop establishment and thus tend to be planted on Medium-Highlands and above, while *boro* rice, which is prepared under wet puddled conditions and transplanted in late January to early February, can be grown on Lowlands and higher relative landscape elevations.

The *kharif*-I *and* II pre-monsoon and monsoon seasons, respectively, span from April through November/December. Transplanted (T.) *aman* rice is Bangladesh’s most common crop, grown during *kharif*-II. The winter *rabi* dry season extends from November/December–April, although *rabi* crops in the southeast of the study area may be sown as late as January. Such late sowing is primairly due to farmers’ preferences for late-maturing *T. aman* rice varieties, and because of late vacating and deep floodwaters on Medium Highland-2, Medium Lowland, and Lowlands (Krupnik et al., 2015a). Where irrigation is available, major *rabi* cereals include *boro* rice, wheat, and increasingly maize. Non-irrigated mustard (*Brassica* spp.), grass pea *(Lathyrus sativus*), lentil (*Lens culinaris*), and mungbean (*Vigna radiata*) are also common, though yields tend to be much lower than what is attainable. Grass pea is often relay seeded into the maturing *T. aman* crop, while lentil is usually seeded as a sole crop, but can also be relayed. Mungbean is conversely sown in late January to early February as a sole crop (Rawson, 2011).

Parts of the study area are embanked by 139 polders constructed in the 1960–70s to protect against seawater intrusion and flooding. Both erosion and intentional embankment breaches are common, which can facilitate irrigation but also increase flood risk (Bernier et al., 2016). The area above the Sundarbans forest in the southwest once experienced southward dry season river flow through the Gorai River, but siltation and the 1976 completion of the Farakka Dam in West Bengal disrupted freshwater flow. This resulted in post-monsoon drying and coastal saltwater intrusion that has now become a serious concern (Brammer, 2002). The south-central hydrological zone is conversely characterized by a dense network of active natural canals and rivers, many of which carry relatively fresher water.

### 2.2 Geospatial analysis

**Fig. 3:**
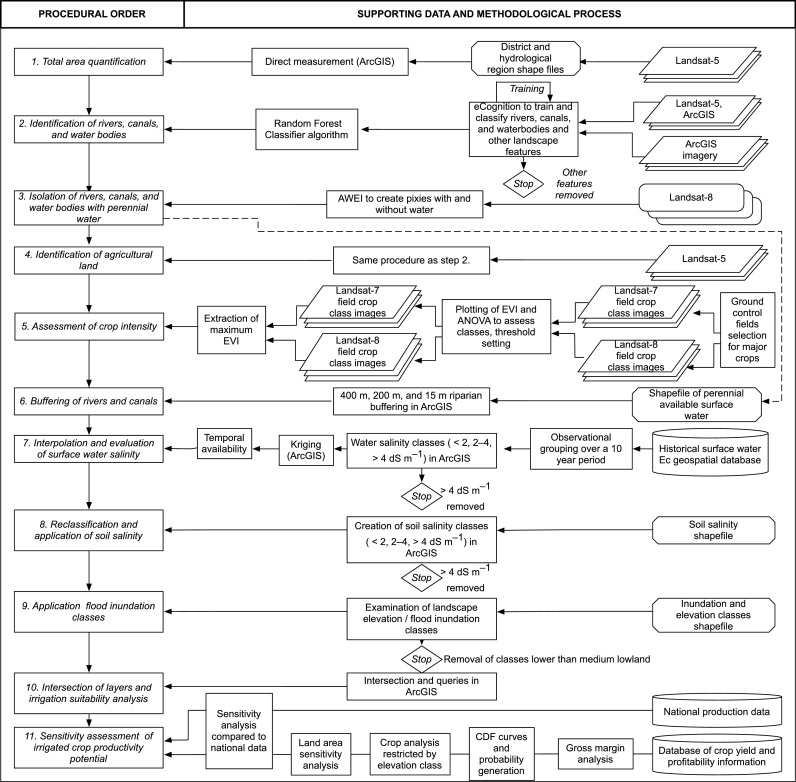
Overview of the methodological procedure and data sources utilized in this paper.

The geomorphology, pedology and hydrology of Bangladesh are extremely complex. Our evaluation of SWI potential therefore followed a multi-step process followed by crop potential scenario analysis (Fig. 3).^1^

#### 2.2.1 Total land area quantification and administrative layers

Available USGS Landsat 5, 7 and 8 images (30 m pixels) were used for analysis (http://earthexplorer.usgs.gov). The study area was covered by Landsat Path 137-Rows 44–45, and Path 138-Row 44.

#### 2.2.2 Identification of rivers, canals, and water bodies

For the identification of rivers, canals, and water bodies, we used Landsat 5 scenes acquired on October 26, 2009 and November 8, 2011, coinciding roughly with the end of the monsoon. We next applied supervised classification to segmented Landsat imagery using eCognition 9 (Trimble Navigation Ltd., Westminster, CO), creating two classes: (1) rivers and canals, and (2) all other landscape features including forest, settlements, transportation networks, and cropland. Training was accomplished using high-resolution satellite imagery of Bangladesh available through Google Earth 7.1.5.1557 (Mountain View, California). Attributes used for segmentation included the mean values of bands 1–5. Texture (all directions) was also used (Haralick et al., 1973). After exporting the resulting shape file with spectral attributes, we employed the Random Forest Classifier algorithm in WEKA (Hall et al., 2009). This created a rule set that was subsequently applied to the remaining segments not used for training. Classification errors were visually controlled and corrected.

#### 2.2.3 Isolation of water networks with perennially available water

Rivers and canals in the study area do not always experience perennial flow. Where they do, their banks may be obscured in satellite imagery by riparian vegetation. For these reasons, atmospherically-corrected 30 m pixel Landsat 8 images from March 21 and 30, 2014 were used to calculate the Automated Water Extraction Index (AWEI). AWEI*_sh_* was used because of its effectiveness in improving water body prediction accuracy under shaded conditions (Feyisa et al., 2014), following Eq. (1):

(1) $$AWEI_{sh}=\rho_{\text{band}2}+2.5*\rho_{\text{band3}}-1.5*(\rho_{\text{band5}}+\rho_{\text{band6}})-0.25*\rho_{\text{band7}}$$

where *ρ* stands for reflectance of the respective Landsat 8 imagery bands, with the output containing pixel classes with and without water.

#### 2.2.4 Identification of cropland

We identified agricultural land as described in Section 2.2.2, though with a third set of Landsat 5 scenes acquired on January 21 and 31, 2010. At this time crop and weed cover is at a minimum, and can therefore be easily distinguished from non-agricultural vegetation. Cropland and non-cropland classes were created, the latter consisting of forest, settlements, infrastructure and transportation networks.

#### 2.2.5 Assessment of crop production intensity

We utilized enhanced vegetation index (EVI) (Huete et al., 2002) measurements to assess crop productivity. EVI is closely related to leaf area index (LAI) and has previously been used in Bangladesh for yield estimation (Schulthess et al., 2012). EVI does not saturate at high LAI and is relatively insensitive to surface soil moisture, making it an ideal measure. EVI was calculated as:

(2) $$\text{EVI}=G\frac{\rho_{\text{NIR}}-\rho_{\text{red}}}{\rho_{\text{NIR}}+C_{1}\times \rho_{\text{red}}-C_{2}\times \rho_{\text{blue}}+L}$$

where G is a gain factor, *ρ* is surface reflectance after atmospheric correction, *C*_1_ and *C*_2_ are coefficients of the aerosol resistance term using the blue band to correct for aerosol derived errors in the red band, and *L* is the canopy background adjustment to rectify differential, nonlinear radiant red and NIR transfer through the crop canopy. Between 10–35 visually-verified and geo-referenced fields of lathyrus, lentil, mungbean, fallow land, *boro* rice, wheat, maize and mustard were selected for EVI extraction in each of the 2011–12, 2012–13, and 2013–14 dry *rabi* seasons from Landsat 7 and 8 imagery acquired between 31 December and 30 March. Twelve Landsat 8 scenes were used to augment the dataset in 2013–14. The near infrared (NIR) band of Landsat 8 has different spectral properties than Landsat 7. Radiance values were consequently cross-calibrated to reflectance using Landsat 7 imagery acquired within eight days of the analyzed scenes. Field number varied across crops because of dissimilarity in year-to-year satellite image coverage and cropping sequences. Fields can also be sown at different dates, resulting in differential phenology even within the same crop species, both within and among years. Repetitive sequential observations were therefore crucial to capture maximum EVI.

EVI values for each crop were plotted as a function of the number of days before or after January 1 until the 100th day of the year. This corresponds roughly to the first two thirds of the dry season, prior to the advent of reliable rains, after which irrigation is less necessary. We grouped each of the cropland types into three production intensity classes. These included (1) fallow land and (2) low production intensity cropland cultivated with legumes (lathyrus, lentil, and mungbean). While these crops can be potentially profitable, they are typically not fertilized, weeded, or irrigated, resulting in yields considerably lower than what could be obtained with more intensive management (Dalgliesh et al., 2011). We therefore classified the remaining cropland as (3) high production intensity, including wheat, *boro* rice, maize, and mustard, all of which are grown with higher levels of intensive management using nutrient inputs and active pest management. Each of the high production intensity crops are typically irrigated with the exception of mustard, which is relay sown into the *T. aman* rice crop.

**Fig. 4:**
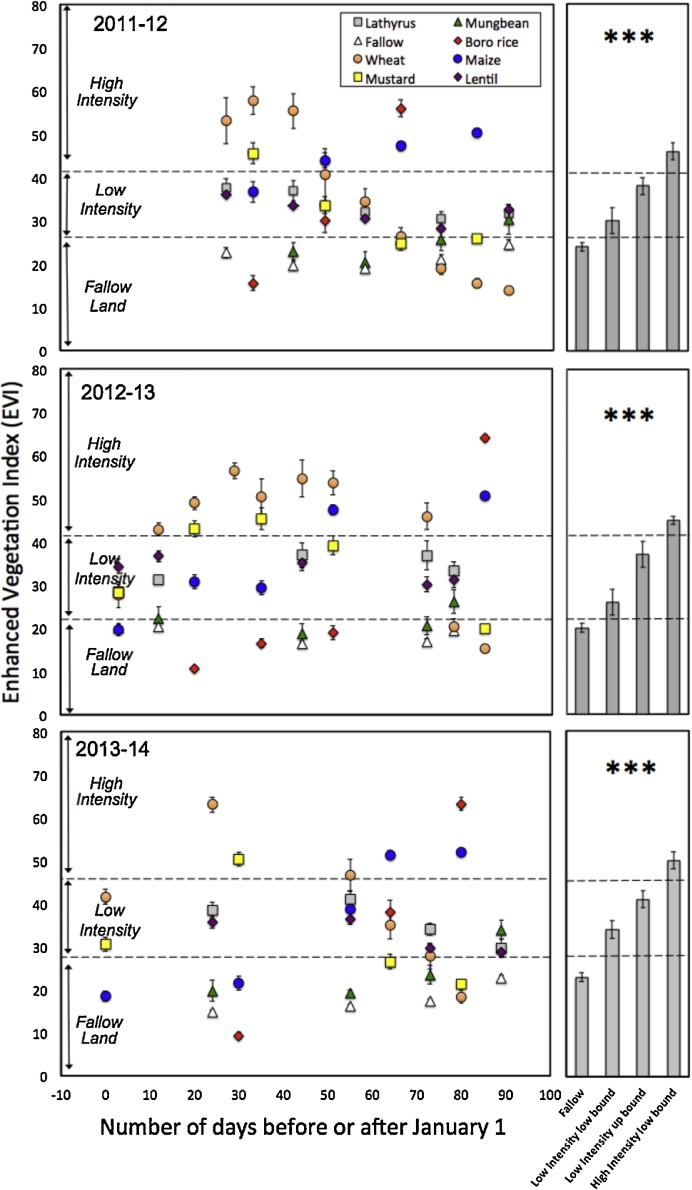
Enhanced vegetation index for predominant crops and fallow lands extracted from calibrated Landsat imagery during the first two thirds of the dry season (−10 to 100 days before or after January 1) in 2012–13, 2013–14, and 2014–15. We classified *boro* rice, wheat, maize, wheat and mustard as high-production intensity crops reliant on irrigation, active pest management, and/or fertilization. Lathyrus, lentil, and mungbean were classified as low-production intensity rain fed crops frequently grown without fertilization, distinguishable from fallows. Error bars indicate standard deviation (SD). Following an ANOVA comparing EVI values for the dates upon which the maximum EVI was observed for these three crop classes in each year, significant differences were detected according to the Tukey-Kramer HSD test at α = 0.05. Threshold upper and lower boundaries between crop production intensity classes were set as the mid-distance point between the lower boundary for the SD of the lowest minimum EVI value and the uppermost boundary of the standard deviation for the highest EVI value between classes. These results are summarized in the grey column charts for each season. *** indicates *P* < 0.001. The dashed horizontal line differentiates each production intensity class. For interpretation of the marker colors in this figure, the reader is referred to the online version of this paper.

Maximum EVI (corresponding to maximum LAI as a measure of peak productivity) for each crop was subjected to a one-way ANOVA using JMP 8.0.2 (SAS Institute Inc., San Francisco), for each of the three dry seasons observed. Separate analysis for each season was necessary because peak EVI varied slightly between years. The *F*-test indicated significance (*P* *<* 0.001) between classes within each season analyzed, with Tukey-Kramer’s range test (α = 0.05) indicating that the fallow, low-intensity, and high-intensity classes were consistently and significantly different (Fig. 4).

We next set EVI-based thresholds used to characterize crop production intensity throughout the study area. Thresholds were set as the mid-distance between the lower boundary for the standard deviation of the lowest maximum EVI observation for the high-intensity cropland types, and the uppermost boundary of the standard deviation for the highest EVI observation for the low-intensity crop use types. For example, in the 2011–12 dry season, mustard exhibited the lowest minimum EVI within the high-intensity crop use category, while the EVI of lathyrus peaked as the highest observation within the low-intensity category. The threshold between high- and low-intensity crop use was therefore conservatively set as the mid-distance between the lower and upper boundaries for the standard deviations of these observations, respectively. This process was also used to distinguish low-intensity from fallow agricultural land use for each season studied. Employing these thresholds, we classified the maximum EVI value for each cropland pixel of the calibrated Landsat scenes for the entirety of the 33,750 km^2^ study area into one of the three crop production intensity classes.

#### 2.2.6 Surface water source buffering

Studies of 12–16 horsepower SWI pumps (Krupnik et al., 2015b) indicate that water can be conveyed from surface sources up to 400 m from river and canal banks where flexible hose piping is used to reduce losses. We therefore created a 400 m buffer around polygons depicting the perennially available water identified in Section 2.2.3. In addition to influencing riverbank erosion, intensive agricultural practices can result in sedimentation and nutrient loading of watercourses. Riparian areas planted with deep-rooted species harness ecosystem services (e.g., nutrient extraction and bank stabilization) to mitigate these negative effects (Power, 2010). We consequently excluded a 15 m strip adjacent to rivers and canals to account for riparian conservation measures.

#### 2.2.7 Freshwater availability: interpolation and temporal evaluation

The regulation of water salinity is an important freshwater ecosystem service that contributes to irrigation potential (Aylward et al., 2005). Salinity concentrations in Bangladesh’s tidal estuary increase as the dry season progresses, a result of the gradual reduction of southward freshwater river and canal water flow after the monsoon. This results in the increased intrusion of brackish oceanic water that can influence surface water salinity and shallow groundwater tables, in turn affecting soil salinity (Brammer, 2013).

**Fig. 5:**
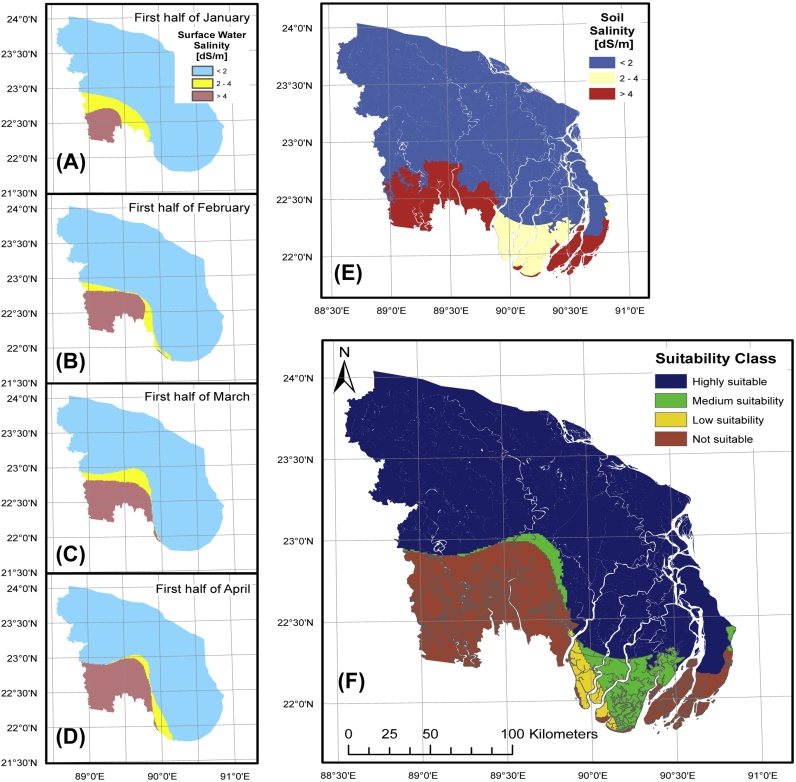
Fresh and brackish water movement in the study zone delta in (A) January, (B) February, (C) March, (D) April. Maximum soil salinity (E) is presented for the height of the dry season before rains start. (F) Combined map overlaying maximum soil and water salinity according to the index detailed in Table 1. For interpretation of the colour classifications used in this figure, the reader is referred to the online version of this paper.

To map the spatial extent of soil salinity and freshwater availability, we assembled surface water salinity readings (*n* = 4,821) from 58 Bangladesh Water Development Board (BWDB) sampling stations. These data covered major rivers and natural canals for the period from 2002 to 2012. We analyzed the first 14 weeks of each calendar year, corresponding to the critical period for dry season crop growth. Observations from each station were grouped into eight half-monthly periods. We then extracted the 90th percentile of these observations as a conservative index for water salinity, and assessed the temporal progression of saltwater intrusion into the delta through Kriging. The resulting maps indicate increasingly poor water quality with the progression of the dry season, especially in the lower southwestern hydrological zone (Fig. 5A–D). Conversely, water quality remains high throughout much of the south-central hydrological zone.

#### 2.2.8 Reclassification of soil salinity

Salinity typically builds during the dry season and is flushed from the soil profile when precipitation commences (Krupnik et al., 2015a). To provide a conservative basis for identifying conditions suitable for cropping, maximum soil salinity measurements at the end of the dry season were utilized. We compared digital maps and metadata for soil salinity in Bangladesh, and selected the SRDI (2000) coastal salinity map as the only resource for which the methodological procedure used to interpolate data was sufficiently clear and robust. This shape file has five ‘mixed’ and eight ‘mostly’ salinity classifications. For example, a ‘mostly’ classification could have included mostly 4–8 dS m^−1^, with some 2–4 dS m^−1^ data. To simplify interpretations, we reclassified data into three classes including <2, 2–4, and >4 dS m^−1^ using the highest reported value for each unit as the basis for reclassification.

#### 2.2.9 Application of flood inundation classes as an indicator of relative elevation

Flood inundation classes provide an indicator of relative landscape elevation which strongly influences crop suitability (Brammer, 2013). Inundation class shapefiles were collected from BARC (2015) and overlaid on the preceding datasets. Lowland and very Lowland, which tend to be poorly suitable for *rabi* cropping, and which were not observed as cultivated in our subsequent crop cut study (see 2.3) were removed.

### 2.3 Layer intersection and salinity index

Shapefiles of dry season water salinity in April were overlaid with those depicting soil salinity to create Fig. 5F. Both this layer and the inundation class shape files were then intersected with cropland polygons. EVI layers within the 400 m buffer (excluding riparian conservation zones) showing surface water availability throughout the dry season were intersected in ArcGIS for the 2011–12, 2012–13, and 2013–14 *rabi* seasons. We then applied a soil and water salinity index to assess the suitability of SWI within the resulting buffered area.

Crop species vary in their ability to withstand soil and water salinity. In order to limit analytical complexity given the range of species included in this study, we created three irrigation water salinity classes based on Ayers and Westcot (1989), including 0–2 and 2–4 dS m^−1^, which we labeled as high- and medium-quality water. Low-quality water >4 dS m^−1^ was assumed to be unsuitable for repetitive rice, wheat, and maize irrigation. The same approach was applied to soil salinity data (Table 1).

Soil and surface water salinity layers were joined with flood inundation land types. This resulted in a geospatial database from which statistics could be extracted regarding the extent of fallow and low-intensity cropland suitable for SWI. We derived the land area falling into each classification by administrative district and hydrological zone, and averaged the results across the three years of observations (Table 2). We also computed the proportion of cropland that could be intensified using surface water irrigation within 10 × 10 km grids imposed over the study area. In accordance with Table 1, non-suitable marginal potential croplands were excluded.

### 2.4 Cereal production scenario analysis

To evaluate potential land productivity resulting from conversion from fallow or low-intensity crops to surface water irrigated maize, wheat, and *boro* rice, we measured yields achieved by farmers on their own fields and in farmer-managed demonstrations implemented by the Cereal Systems Initiative for South Asia (CSISA) project. These three crops are among the most important cereals grown in Bangladesh from a food security and income generation perspective. Crop cut measurements and data were collected throughout the study zone during each dry season from 2011 through 2015. In total, 510, 550, and 553 irrigated wheat, maize, and rice crop cuts were made. Yields were obtained from a minimum of 10 m^2^ and were moisture corrected.

We collected additional data from farmers on sowing dates and the labor and monetary requirement for all major crop management operations. These included costs for land preparation, sowing, pest management, fertilizer and irrigation application, as well as harvest activities. Prices for inputs and grain and straw outputs were assigned as the average of three observations from independent dealers and buyers in local markets. These data were combined with farmers’ reported local wage rates to calculate labor costs. We then conducted cost-benefit analyses for each farmer and crop observation, converting Bangladesh Taka (BDT) at 77 BDT to 1 USD.

Farmers’ observed yields and profits were next geospatially categorized according to the soil and water index in Table 1. Classified yield and gross margin data were then plotted as cumulative distribution functions (CDFs) to identify each crop’s performance in high and medium potential index classes at the 25th, 50th, and 75th percentiles. Based on observed data, these percentiles are equivalent to *P* = 0.75, *P* = 0.50, and *P* = 0.25, respectively (Hardaker et al., 1997). In other words, the CDF shows the probability that a variable will take a value equal to or less than *x* on the abscissa. Our use of CDFs therefore provides a simple method to depict the inter-quantile range of potential production scenarios.

The land upon which dry *rabi* season crops can be grown in Bangladesh is partially governed by monsoon season flood inundation classes that are indicative of their relative landscape elevation, as lower lying land may drain too late to establish dry *rabi* season crops on time. Wheat is generally suited to Medium-Highland-1 and −2, and higher classes (Krupnik et al., 2015a). Maize, which is sown at similar times, is usually established at the same relative elevation classes. Our data indicated that maize or wheat farmers never established crops on elevation classes lower than these. Observations of *boro* farmers conversely showed that Medium-Lowland and higher land classifications were used for rice, while Lowlands with >180 cm of monsoon season flooding were not.

Modeling the potential for intensification, we assumed that some land that could be double cropped with irrigation would remain in rainfed legume cultivation. We therefore modeled potential productivity with scenarios in which wheat, maize, and *boro* rice were simulated as if they were established on 25%, 50%, and 75% of the three-season averaged fallow and low production intensity cropland identified as suitable for SWI. Production and profitability estimates were then constructed with data from the CDFs at *P* = 0.25, 0.50, and 0.75 for each resulting land area, but restricted to Medium-Highland-2 and above land types for wheat and maize. *Boro* rice was conversely subjected to the same constraints, but analyzed on all relative landscape elevation classes excluding Lowlands and very Lowlands, in line with our observations of locations where farmers established rice. The resulting range in aggregate yield results were then assessed in terms of their potential contribution to national maize, wheat, and *boro* rice production by comparing them to BBS (2013) statistics for 2011–12, the most recent year for which country-wide production data were available.

## 3 Results

### 3.1 Total agricultural land area and crop intensity assessment

Out of 1.93 million ha of agricultural land in the study zone, 12% and 45% were identified as fallowed and under low production intensity rainfed crops, respectively, during the *rabi* season (Table 2). In the south-central hydrological zone, most of this land was concentrated in Patuakhali and Barguna districts.

### 3.2 Conversion potential for surface water irrigation

Considering land within 400 m of surface water as reachable by irrigation pumps, but also discounting area within 15 m of watercourses for conservation purposes, 47,066 and 132,470 ha were identified as fallow and rainfed low production intensity cropped land, respectively (Table 3). According to the soil and water salinity index (Table 1), 21% of the observed fallows were classified as high and medium potential, with the remainder poorly suited to SWI. Conversely, 78% of the observed low production intensity acreage addressable by SWI was classified as high and medium potential.

**Fig. 6:**
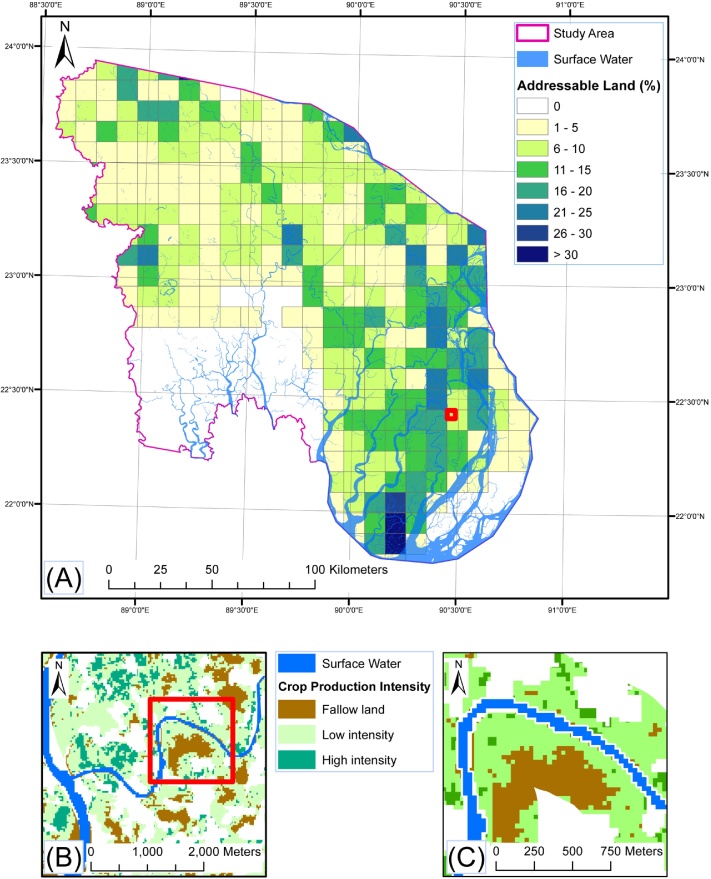
(A) Agricultural land suitable for surface water irrigation expressed as percentage of total cropland area in 100 km^2^ imposed grids. Low- and martinal potential croplands were excluded. (B) Detail of the red inset in Fig. 6A depicting a block of low-production intensity and fallow land proximal to surface water. (C) Further detail depicting the 385 m buffer indicating precise locations of fallow and low-intensity cropland upon which surface water irrigation could be used. Areas in white are non-agricultural land uses. Much of the white area adjacent to surface water in figure section C depicts the riparian buffer retained in our analysis to for conservation purposes. For interpretation of the references to colour in this figure legend, the reader is referred to the online version of this article.

The largest concentration of fallow land that could be brought under SWI was located in the south-central hydrological zone (Fig. 6A–C), including 8,771 and 6,361 ha under high and medium potential soil and water salinity index classifications (Table 1). Fifty-nine percent was located in Barisal Division, the majority in Patuakhali (Table 3). The same district however also had the highest amount of low and marginal potential land, a result of increasing salinity towards the coast (Fig. 5F).

As with fallow land, most (73%) of the low production intensity cropland that could be converted from rainfed to irrigated conditions was found in the south-central hydrological zone. Within this area, Patuakhali district had the greatest land area where soil or water salinity exceeded 4 dS m^−1^, indicating low or marginal SWI potential. Crops with an EVI between 25 and 43 were detected in these areas, indicating the presence of rainfed legumes. In these environments, farmers frequently cultivate legumes using residual soil moisture following rice harvest, supplemented by occasional precipitation, although yields are typically low (Dalgiesh and Poulton, 2011). Similar low production intensity land was detected in the central belt of the south-western hydrological zone. In this area, saline groundwater intrusion and intensive brackish aquaculture production have resulted in increased salinity concentrations north of the Sundarbans forest reserve (Table 3 and Fig. 5A–D).

### 3.3 Irrigated crop productivity characteristics

Across all years of observed data, the range of sowing dates for wheat was November 10 to December 24 (mode: November 21). The range for maize was November 9 to January 28 (mode: January 4). For both crops, later sowing tended to occur in Barisal Division. The range of sowing dates for *boro* rice was November 24 to January 10 (mode: January 6). Rice was transplanted 34–68 days after sowing (mode: 42). Wheat farmers applied 1–4 irrigations, and between 0 and 245 kg N, 0–89 kg P, 0–152 kg K, 0–51 kg Ca, 0–42 kg S, and 0–2 kg Zn ha^−1^. Maize farmers also applied 1–4 irrigations, but with 107–258 kg N, 10–78 kg P, 13–163 kg K, 0–68 kg Ca, 0–56 kg S, and 0–3 kg Zn ha^−1^. *Boro* rice farmers conversely applied irrigation 6–22 times, with 36–171 kg N, 0–39 kg P, 13–101 kg K, 0–30 kg Ca, 0–25 kg S, and 0–3 kg Zn ha^−1^. Farmers’ use of organic amendments and use of pesticides were limited.

**Fig. 7:**
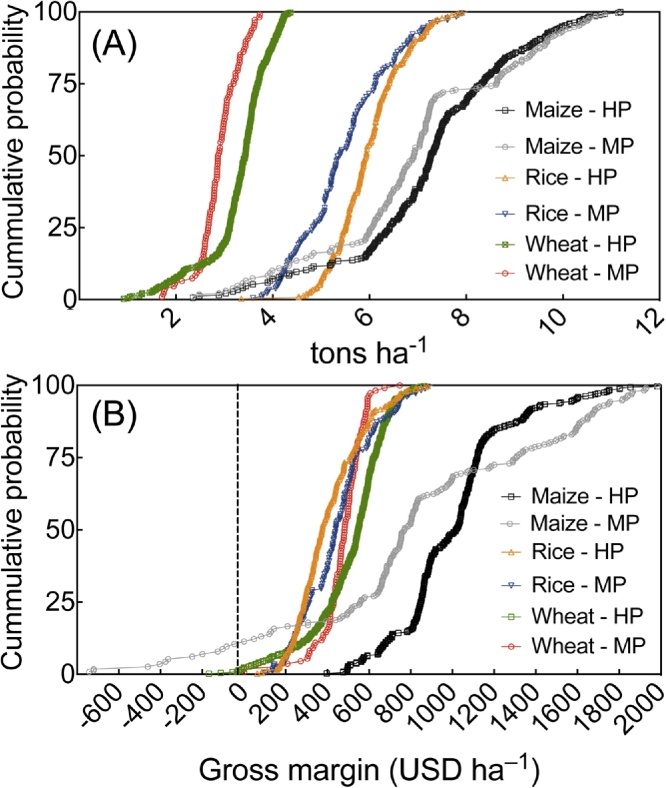
Descending cumulative distribution function (CFD) for (A) irrigated maize, *boro* rice, and wheat yields (t ha^−1^) and (B) gross margins (USD ha^−1^) obtained by farmers on high (HP) and medium potential (MP) land considering the soil and water salinity index (Table 1) between 2011 and 2014. *n* = 321 and 229 HP and MP observations for maize; 375 and 179 observations for rice, and 416 and 108 observations for wheat, respectively. For interpretations of the colour markers used in this figure, readers are referred to the online version of this paper.

The 25th, 50th, and 75th percentiles of the CDF for wheat yields were 3.11, 3.42, and 3.96 t ha^−1^, respectively, for observations identified within the high-potential water and soil salinity classification. In medium-potential areas the same percentiles indicated wheat yields of 2.67, 2.87, and 3.19 t ha^−1^ (Fig. 7A). Resulting gross margins were 422, 548, and 625 USD ha^−1^ for under the high-potential classification (Fig. 7B), and 431, 483, and, 539 USD ha^−1^ in medium-potential classifications. Farmers growing *boro* showed slightly poorer performance when comparing medium to high-potential soil and water classifications, e.g, 4.83 vs. 5.11, 5.39 vs. 5.93, and 6.12 vs. 6.41 t ha^−1^ for the same percentiles, corresponding to gross margins of 290, 376, and 508 USD ha^−1^ for high-potential land, and 314, 440, and 536 USD ha^−1^ for medium-potential land. The quartile range of maize yields achieved on medium-potential land were 6.05, 6.91, and 8.63 t ha^−1^ at the 25th, 50th, and 75th percentiles; on high-potential soil and water classifications, observed values were 6.42, 7.27, and 8.32 t ha^−1^. Maize profits were on average 547 and 460 USD ha^−1^ greater than *boro* rice and wheat, respectively. Higher profits resulted from higher maize yields and favorable market prices, although storm induced crop lodging resulted in poor crop performance and negative returns for ten farmers on medium-potential land in Barisal (Fig. 7B).

### 3.4 Production scenario analysis

We analyzed the aggregate production potential of wheat, maize, and *boro* rice were they to be established on the combined fallow and low production intensity cropland identified as suitable for conversion to SWI. This was accomplished through scenario analysis using observations from the inter-quartile range of yield data from the CDFs. Based on these distributions, our yield data are reliable at probability levels equivalent to 0.25, 0.50, and 0.75. The resulting data were applied to one-quarter, one-half, and three-quarters of the observed fallow and low-intensity cropland within buffer areas, in order to leave additional area open for alternative land uses. Our estimates however assume full coverage by rice, wheat, or maize in each independent scenario of one- to three-quarters coverage. In reality, farmers would likely cultivate a mixture of cereals and other crops, requiring optimization modeling to assess and target the ‘best-bet’ configuration of crop allocation for farm-level yields and profits, a subject beyond the scope of this paper. For this reason, we conversely present scenario ranges to delineate the size of the potential production envelope for maize, *boro* rice, and wheat on an individual basis.

Considering the more conservative 75th probability level for yield, the estimated aggregate production potential for maize ranged from 166,659–499,976 tons within one season, assuming the crop were planted on one- to three-quarters of the buffered land area suitable for SWI (Table 4). Were the same areas established with wheat at the same probability level, an estimated 85,671–257,012 tons could be produced, while the range of production for *boro* rice was 167,659–502,977 tons. Our least conservative estimate used data from the 25th probability level, resulting in an estimated range of 237,729–713,188 tons of maize, 101,648–304,994 tons of wheat, or 198,126–595,381 tons of *boro* rice on one- to three-quarters of the buffered land area. Regardless of the estimated coverage area, the majority of the land that could be cultivated using SWI would come from conversion of low production intensity rainfed cropland. Modeling the potential economic consequences of SWI, we estimate that farmers could generate between 9.07–108.2 million USD (at *P* = 0.25), on one- to three-quarters of the buffered land, depending on the crop chosen, with the order of profitability ranges following maize > wheat > *boro* rice at all probabilities between 0.25–0.75.

### 3.5 Potential contribution to national aggregate cereals production

During the winter of 2011–12, the most recent season for which national level data are available, a total of 1.72 million tons of maize were produced in Bangladesh, from 232,000 ha of land. Roughly 0.99 million tons of wheat were also produced from 258,000 ha, while *boro* production was considerably greater, at 18.76 million tons from 4.81 million ha (BBS, 2013). Both the land area and aggregate yield of maize and *boro* rice were the highest in this season compared to the previous ten-year period. Conversely, wheat area and aggregate yield reached a peak in 1998–99 at 882,000 ha and 1.91 million tons produced, with slow decline thereafter, largely a result of land conversion to maize.

**Fig. 8:**
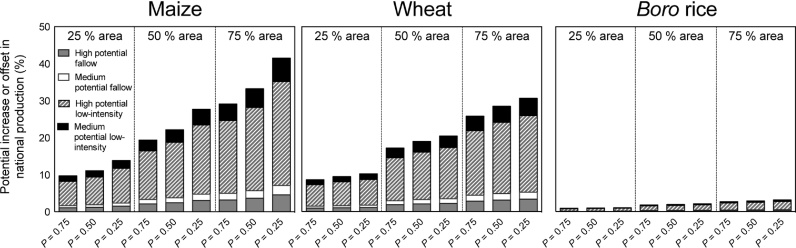
Modeled results describing the potential contribution of surface water irrigated (SWI) *boro* rice, wheat, and maize to national aggregate production in Bangladesh. Data depict three high and medium suitability land coverage scenarios with respect to the index shown in Table 1, including projections using one-quarter, one-half, and three-quarters of all fallow and low-intensity crop land identified within riparian buffers. Probability values indicate the probability of contribution from SWI cereals at *P* *=* 0.25, 0.50, and 0.75 derived from the CDFs (Fig. 7).

Utilizing the aggregated data estimates data in Table 4, we modeled the potential contribution of each crop grown using SWI in the study area to national cereals production in Bangladesh. Although 167,659–594,381 more million tons of *boro* could be produced on the identified SWI suitable land in the study area, the potential contribution to national production never exceeds 3.2%.This observation is a result of the large quantity of *boro* already produced in Bangladesh. Cultivation of maize on one- to three-quarters of the area identified as suitable for SWI would contribute between 10 and 29% more maize nationally at the 75th probability level, or 14%–42% at *P* = 0.25 (Fig. 8). Considering wheat, between 9% to 26% more could be produced from the same area at *P* = 0.75, or 10%–31% at *P* = 0.25.

## 4 Discussion

The achievement of double cropping is an important development objective for the GoB, with USD 500 million of future donor investments earmarked for surface water irrigation initiatives (MOA and FAO, 2013). We studied the spatial distribution of fresh surface water availability and crop production intensity in a 33,750 km^2^ case study area to evaluate the potential of conversion from fallow or rainfed cropland into intensified cereals production using decentralized SWI. We conservatively estimated that there are 20,800 and 103,000 ha of fallow and rainfed cropland that could be brought into double cropping, with considerable potential production gains for maize and wheat. Land use planning policy in Bangladesh conversely emphasizes *boro* rice to offset increasingly high-energy subsidy and production costs in the country’s intensively groundwater irrigated areas (Bell et al., 2015, MOA and FAO, 2013, Qureshi et al., 2015). Our data however indicate that it is unlikely that decentralized SWI could augment national rice production (or conversely offset it in groundwater irrigated areas) at levels above three percent of recent national production. These results therefore have important implications on agricultural land use policies, casting doubt on the often made assumption that SWI expansion in the south of Bangladesh can replace groundwater irrigated *boro* production in the north of the country.

The earliest remote sensing attempt to assess land fallowing for intensification in southern Bangladesh was performed by Poulton (2011), who estimated that 230,000 ha – 4% greater than our assessment – were fallowed during the 2006–07 *rabi* season.. Their study however relied on extrapolation from a smaller 1,592 ha case-study area to all of southern Bangladesh. More recently, Gumma et al. (2016) used 250 m resolution MODIS satellite imagery to estimate rice-fallow rotational area in South Asia that could be converted to rice-legume cropping systems. Their results indicated that 8.7% (1.9 million ha) of the region’s *rabi* season fallowed land is found within Bangladesh, though data were not disaggregrated within country. Others have used survey methods to assess land use and crop production intensity. BADC (2010) reported that 634,000 dry season hectares are regularly fallowed in southern Bangladesh, while the Bangladesh Bureau of Statistics, or BBS (2013) estimated 240,000 ha. The Ministry of Agriculture (MoA) and FAO (2014) however placed the number at 136,000 ha. The current paper, however, is the first attempt to map rainfed and fallow land, while also applying biophysical data on land and water suitability, and combining them with a large-scale crop-cut network to assess cereals intensification potential through surface water irrigation.

The discrepancies in fallowed land reporting in the above sources may be due to conflicting notions of what constitutes a sufficiently low level of production intensity to qualify as fallow. Although legumes can be considerably profitable, farmers typically cultivate broadcast pulses as an ‘opportunity crop’, growing them without inputs and using only residual soil moisture, precipitation, and minimal management (Rawson, 2011). Such studies may misclassify such extensive management as ‘fallow’. Further inaccuracy may also result from inadequate sampling frameworks. According to Poulton and Dalgliesh (2008), Bangladesh’s Department of Agricultural Extension performs quarterly surveys of five large to small-scale farms at the union (lowest administrative) level, combined with cluster-plot rather than random or spatially explicit sampling to estimate total cropped area. The BBS uses a similar approach and subsequently scales these data to the district level, while also conducting separate crop-cut surveys. The dynamic nature of cropping in Bangladesh also poses analytical problems, as fields may be fallow one year but cropped the next. To overcome this constraint, we integrated three years of 30 m pixel Landsat observations to develop a more robust index of crop production intensity.

The largest blocks of fallow and low production intensity crops that can be addressed with quality surface water for irrigation are found in the south-central hydrological zone, in the north of Patuakhali and Barguna, and in Barisal district. Compared to the southwest, this zone benefits from active river flow that mediates dry season estuarine saltwater intrusion, while partially regulating groundwater and soil salinity (Brammer, 2010, Brammer, 2013, Rawson, 2011). This area is nonetheless among the IGP’s poorest (World Bank, 2010), with farmers’ current fallowing practices and extensive approach to dry season cultivation indicating that population and land pressure, as well as market opportunities, still remain below the threshold at which intensification may independently develop (cf. Turner and Ali, 1996).

While our analysis focused on the biophysical constraints and opportunities for increased crop productivity, the successful use of surface water irrigation for crop intensification has other socioeconomic barriers that require consideration. Poor water governance in the semi-saline coastal fringe is particularly relevant. Conflicts over water resources allocation have however been more severe in Khulna and Satkhira districts on the western half of the study area, where *boro* rice farmers compete with wealthier aquaculturists who encourage intrusion of brackish water for prawn production (Bernier et al., 2016, Dewan et al., 2014). Our analysis however indicated that the greatest potential for surface water irrigation is in Barisal and Patuakhali districts, in the south-central hydrological zone, which has comparatively little history of water resources conflict. Appropriate water governance policies are nonetheless likely to be an important prerequisite to build an environment in which intensified cropping is possible at a large scale. Improved grain storage facilities and strengthened input and output markets are also prerequisite to sustainable intensification.

Periodic flooding and cyclonic storms in the southern region are anticipated to accelerate with climate change (IPCC, 2014). Combined with high poverty levels, these factors exert a strong influence on vulnerability in coastal Bangladesh, highlighting the importance of adaptation and risk-reduction measures (Akter and Mallick, 2013). Shareholder cropping is also common, as are labor shortages from increasing seasonal migration where farmers seek more remunerative urban employment (MOA and FAO, 2013, Zhang et al., 2014). Wages earned through non-agricultural labor and remittances are an important source of revenue in Bangladesh, and are commonly re-invested in rural communities, which supports their development (Zhang et al., 2014). Turner and Ali (1996) demonstrated that farm labor demand and drudgery limits smallholders’ interest in increasing production beyond immediate household needs in Bangladesh. This highlights the need for policy supporting farm mechanization options appropriate for smallholder farmers, which can decrease labor demand while improving the precision of agricultural operations (cf. Krupnik et al., 2013). Programs supporting under-represented, tenure-insecure farmers are also likely to be necessary to address equitable development.

While our analysis focused on the potential for decentralized SWI provided by pump owners servicing small blocks of farmers, much of the SWI investment called for by the GoB is aimed at large-scale irrigation schemes in which farmers’ participation is assumed on an *a priori* basis. Experience has however shown that such schemes can entail engineering and managerial inefficiencies. Combined with farmers’ lack of interest, suboptimal scheme performance, and low returns on investment have been observed (Brammer, 2002, Brammer, 2010). The Barisal Irrigation Project, initiated in the early 1980s, was for example intended to bring 42,000 ha into production. Only 10,000 ha were however achieved. Poor administration and farmers’ preference for non-*boro* crops caused friction with water managers, which negatively influenced performance (Brammer, 2002). The 72,000 ha Ganges-Kabadak project, initiated in 1955, was intended to provide flood protection, internal drainage and irrigation, although most of the allocated land was already double-cropped and thus contributed little to intensification (Brammer, 2002). In these schemes, farmers were initially asked to rent low-lift water pumps from the State, though farmers’ lack of autonomy resulted in their unwillingness to invest in SWI (Brammer, 2010, Hossain, 2009).

Conversely, the ‘irrigation boom’ (*sensu* Shah, 2009) that spurred the expansion of double cropping in northern Bangladesh was achieved not through centralized schemes, but rather by hundreds of thousands of decentralized and small-scale entrepreneurs operating independent pump sets to provide shallow groundwater to farmer-clients (Palmer-Jones, 2001, Qureshi et al., 2015). Today, over 90% of Bangladesh’s irrigation is supplied in this way (Chowdhury, 2010). Similarly decentralized irrigation supplied by service providers operating portable low-lift pumps and managing smaller command areas mimics this approach, and may be more feasible for expanding SWI (Krupnik et al., 2015b). Measures to rationalize abstraction will nonetheless be crucial to maintain freshwater availability in the coastal fringe (Bell et al., 2015, Bernier et al., 2016)

Bangladesh has achieved near rice self sufficiency using groundwater irrigation in the country’s north (Hossain, 2009, Mainuddin and Kirby, 2015). The GoB’s emphasis on SWI conversely stems from policy priorities to shift from subsidy- and energy-intensive groundwater irrigation to less costly production modalities using surface water in the south (MOA and FAO, 2013, Qureshi et al., 2015). Shifting *boro* rice production southward is nonetheless controversial, and may not be as profitable or feasible as anticipated (Bell et al., 2015, Bernier et al., 2016, Qureshi et al., 2015).

Accounting for national-level production of pre-monsoon *aus* and monsoon season *T. aman* rice in addition to *boro*, Bangladesh produced over 32 million tons between 2011 and 2012 (BBS, 2013). Using this as a base, our scenario analysis indicates the potential annual contribution from surface water irrigated *boro* in the study area to national rice production ranges between just 0.5% to a maximum of 1.9%. This comes despite the larger potential production area (3,480–17,665 ha) that included rice grown on Medium-Lowlands, whereas maize and wheat were conversely never established below the Medium-Highland elevation class by the farmers observed in our dataset. *Boro* rice can also be grown on Lowlands, though none of the farmers in our dataset did so. Yet if the remaining high- and medium-potential Lowland area identified by our analysis were added to our simulations, another 7,842 ha of boro rice could be grown in the study area. Aggregate national *boro* production would however increase by only 0.06-0.20% compared to our estimates without Lowlands.

The energetics and environmental ramifications of surface water withdrawal also require critical reflection. Our field observations showed *boro* rice farmers using 6–22 irrigations, compared to 1–4 for wheat and maize. High irrigation rates can be costly, even with relatively more efficient surface water pumps (Krupnik et al., 2015b). Our generally lower observations of *boro* profits were a result of greater proportional irrigation costs, which constituted the largest single expense at 18% of rice farmers’ total production costs, followed by fertilizers (13%). The potential for excessive freshwater withdrawal to affect biodiversity and/or to disrupt the ecosystem service of estuarine saltwater regulation has also been raised (Brammer, 2010, Chowdhury, 2010). Increased surface water abstraction from smaller canal systems with limited flow is also a concern, as heightened water scarcity may result. Further research is needed to define environmentally safe abstraction levels, especially in light of the anticipated effects of climate change and sea level rise (Bell et al., 2015).

The importance of rice in the Bangladeshi diet however indicates a strong likelihood that farmers would nonetheless respond to greater SWI availability with increased *boro* production, although crop diversification also has important role to play in rationalizing SWI investments. Seventeen percent of the average Bangladeshi citizen’s diet is comprised of wheat, at 25 kg person^−1^ year^−1^. Domestic wheat supply is however less than half of national requirements (Mainuddin and Kirby, 2015). Wheat’s low irrigation requirements position it as an energy efficient crop in comparison to *boro*, with implications for reducing subsidy expenditures (Qureshi et al., 2015). The crop’s shorter field duration also means that it can be planted and harvested before the onset of intense pre-monsoon storms that can cause flooding and crop lodging. Because wheat yield is negatively correlated with sowing date (Krupnik et al., 2015a), further risk-reducing measures are possible where post-monsoon drainage can be supplied to advance sowing dates.

Conversely, emerging threats such as wheat blast disease (*Magnaporthe oryzae Triticum*pathotype), which appeared in Asia for the first time in 2016 and affected 15% of Bangladesh’s wheat area, must also be considered (Malaker et al., 2016). Though blast inflicted yield losses were highly variable, Islam et al. (2016) reported a 25% average yield reduction measured across 100 farmers in eight districts. Applying this percentage loss to our dataset, the potential contribution of surface water irrigated wheat to national production would decline by 21,420–76,230 tons across the scenarios analyzed, representing a potential loss of 2%–8% from our baseline scenarios. Although the risk of blast reoccurrence is currently uncertain, the threat posed by the pathogen underscores that action is urgently needed to develop and deploy integrated pest management measures.

Maize production in Bangladesh is also 80% below the country’s estimated demand of 1.2 million tons year^−1^ (Uddin et al., 2010). An increasingly important market crop, maize offers opportunities for income generation when sold as poultry or fish feed stock, while also requiring only limited irrigation (Qureshi et al., 2015). Bangladesh’s cropped maize area has increased by 98% since 1990, largely by supplanting *boro* rice in the country’s north (BBS, 2013). Maize is however a tall-statured and long duration crop, and is hence at risk of lodging and storm damage in the coastal south (Akter et al., 2016). Efforts are therefore needed to develop high yielding but shorter duration cultivars suitable for the study area.

Salinity tolerant crop genotypes are also important, as nearly one-quarter of the observed fallow and low-productivity addressable land fell into the medium-potential category. Although maize and rice are less tolerant, wheat is capable of withstanding water salinity up to 3.8 dS m^−1^ for pre- or post-sowing irrigation before yield declines (Ayers and Westcot, 1989). Continued application of semi-saline irrigation on soils with shallow groundwater tables, such as in the coastal fringe, could however degrade soil quality and shift their status from medium- to marginal-potential over time. A significant amount of salt may still be flushed from the root profile during the monsoon (Rawson, 2011). Additional efforts are thus required to model the most appropriate allocation of surface water of varying quality to irrigated crops on different land types and salinity levels. By integrating these efforts with information on the proximity of markets and grain prices, bio-economic decision support tools could be developed to advise policy makers, development planners, and farmers on the best-bet mixture of crop species diversity to reduce risk, provide for food security, and increase incomes. In areas with soil and water salinity >4 dS m^−1^, shifting from crops to aquaculture may however be a more viable land use strategy, as indicated by the rapid growth of intensive prawn production near the coast of the southwestern hydrological zone (Bell et al., 2015).

Bangladesh’s estuarine delta is complex; combined with the limitations of publically available data, our work should be interpreted cautiously. Much of the coastal fringe is enclosed by polders, some of which experience post-monsoon waterlogging that delays *rabi* season crop establishment, resulting in suboptimal crop performance. Our observations of irrigated cropping were made in areas lacking drainage, though farmers achieved relatively acceptable ranges of sowing dates, yields, and profitability levels. Earlier sowing can have a strong effect on yield (Krupnik et al., 2015b); and as such, further geospatial assessment is required to prioritize locations that could be intensified with decentralized SWI, and which would benefit from drainage and improved sluice gate engineering.

While we used an automatic water extraction index to identify the availability of surface water, there are currently no reliable remote sensing methods to measure the depth and hence volume of observed water resources. Publically accessible data from a sufficiently dense grid of georeferenced and manually observed river depth observations are also lacking. Physical water scarcity can both hamper and result from surface water irrigation, particularly in smaller and silted canals, though additional research is needed to map and confirm this hypothesis. Conversely, the water extraction index utilized 30 m pixel Landsat imagery such that we were able to detect only wider rivers and canals. Southern Bangladesh has a considerable number of poorly mapped canals less than 30 m wide, indicating that more surface water resources (and hence irrigable fallow and rainfed cropland) may be available than detected in our conservative analysis. We identified the most promising area for intensification as the south central hydrological zone. This area is more hydrologically active than the west of the study area, where upstream damming of the Ganges and shifting channel bed formation causes reduced flow and increased salinity intrusion in the Gorai river network (Brammer, 2013). Lastly, current salinity surveillance lacks sufficient water sampling at the far southern tip of Patuakhali, which may result in slight overestimation of medium-suitability area.

## 5 Conclusions

The Government of Bangladesh has emphasized sustainable intensification in southern Bangladesh as an important land use policy objective, and is planning considerable investment in surface water irrigation to meet this aim. The desired outcome of these efforts is to move rice-fallow and rice-rainfed crop farmers into irrigated rice–rice double-cropping, in order to shift *boro* rice from the country’s north where production requires overly costly energy subsidies, and to keep pace with increasing national demand. The planned approach focuses on the construction of large-scale, state-sponsored surface water irrigation schemes, although similar approaches have previously had disappointing results. Appropriate location of irrigation facilities is also a concern given soil and water salinity constraints, in addition to the spatial and temporal heterogeneity of quality water availability.

This research responded to these problems by examining an alternative model − decentralized surface water irrigation – to augment *boro* rice, wheat, and maize production on seasonally fallowed and rainfed cropland. To accomplish this aim, we integrated remotely sensed observations of crop production intensity with georeferenced environmental data, including assessment the ecosystem services of salinity regulation and freshwater provision, in order to identify and target where irrigation has the most potential to encourage intensification. We combined this effort with crop cut observations from irrigated rice, wheat, and maize farmers, and performed scenario analyses to assess the potential contribution from surface water irrigation to national cereals productivity.

Our findings indicate substantial scope for decentralized surface water irrigation in southern Bangladesh, even in the face of soil and water salinity constraints, although the potential for *boro* production appears to be more limited than anticipated. Dry season cultivation of wheat or maize cropping could however result in significant production increases, with implications for national food security, while also addressing income generation and minimizing water withdrawals and environmental risks. Our results should however by interpreted cautiously. Further studies into alternative crop and land use options, and best-bet policy mechanisms to align risk reduction, finance provision, market access, and improved water governance are still needed. In northern Bangladesh, dry season irrigation initially expanded through relatively unrestrained shallow tube well installation, resulting in unsustainable energetic costs and pressure on groundwater in intensively cultivated areas. Proper environmental monitoring and conservation policy only began to be adopted after resource extraction problems became evident. Learning from these lessons, surface water irrigation and intensification efforts will require measures to manage withdrawal, especially as the ecosystem services of salinity regulation and freshwater provision are reliant on the maintenance of sufficient southward flow. These issues aside, our results support the targeted use of surface water irrigation, in partial support of Governmental land use policies favoring rice production, though crop diversification options should be seriously considered.

## Figures and Tables

**Table 1:** Soil and water salinity analytical matrix used to filter cropland potential for dry rabi season intensification using surface water irrigation.

Salinity (dS m^−1^)			
Soil	Water		
	0–2	2.1–4	>4
0–2	High potential	Medium potential	Marginal potential
2.1–4	Medium potential	Low potential	Marginal potential
>4	Marginal potential	Marginal potential	Marginal potential

*Note*: land identified as marginal potential was excluded and not considered in our analysis.

**Table 2:** Total land area, agricultural and non-agricultural land area (excluding areas used for aquaculture), andm agricultural land production intensity classification in the study area in southwestern Bangladesh. Data are derived from Landsat 7 and 8 and are the averages of the 2011–12, 2011–2013 and 2013–2014 *rabi* seasons set by thresholds identified in Fig. 4.

Division	District	Hydrological zone^a^	Total land area (ha × 10^c^)			Agricultural land production intensity classification (ha × 10^c^)		
			Total land area	Cropland area	Other^b^	Fallow land	Low production intensity cropland	High production intensity cropland
Barisal	Barguna	SC	131	93	38	16	63	14
	Barisal^c^	SC/SW	219	106	113	4	43	59
	Bhola	SC	155	78	77	12	35	30
	Jhalokati	SC	72	37	35	3	23	11
	Patuakhali	SC	246	169	78	34	113	21
	Pirojpur	SC/SW	121	61	60	5	39	17

		*Sub-Total*	*943*	*543*	*400*	*74*	*316*	*153*

Chittagong	Chandpur^c^	SC	7	3	5	0	1	2

		*Sub-Total*	7	3	5	0	1	2

Dhaka	Faridpur^c^	SC/SW	200	126	75	13	68	44
	Gopalganj	SC/SW	147	113	34	6	35	73
	Madaripur^c^	SC/SW	114	71	43	3	21	47
	Manikganj^c^	SC	8	4	4	1	2	1
	Rajbari^c^	SC/SW	102	66	36	9	42	15
	Shariatpur^c^	SC	99	53	46	1	15	37

		*Sub-Total*	*671*	*433*	*237*	*33*	*183*	*218*

Khulna	Bagerhat	SW	203	77	126	15	37	25
	Chauadanga	SW	114	86	28	3	29	53
	Jessore	SW	258	172	85	9	57	107
	Jhenaidah	SW	197	151	46	6	69	76
	Khulna	SW	207	106	100	53	28	26
	Kushtia^c^	SW	74	57	17	4	28	25
	Magura	SW	105	75	30	6	42	27
	Meherpur^c^	SW	70	51	19	2	15	33
	Narail	SW	98	77	22	10	36	31
	Satkhira^c^	SW	217	95	122	27	25	43

		*Sub-Total*	*1542*	*947*	*595*	*133*	*367*	*446*

		Total area	3163	1926	1236	240	867	819

a SW = South-western; SC = South-central. Note that some zones bisect select districts.

b Includes forest, habitation, road networks, industrial, and other areas.

c Districts that are not entirely covered by the area of interest.

**Table 3:** Range in agricultural land area and suitability classification for surface water irrigation within the 400 m buffer of rivers, canals, and other dry rabi season available water sources, under fallow and low crop productions intensities during the dry rabi season based on Landsat-5 observations averaged across the 2011–20112, 2012–20113, and 2013–2014 *rabi* seasons in southern Bangladesh^a^.

Division	District	Hydro zone^b^	Agricultural land use intensity and suitability classification							
			Fallow land (ha)				Low-production intensity crop land (ha)			
			High potential	Medium potential	Low potential	Marginal	High potential	Medium potential	Low potential	Marginal
Barisal	Barguna	SC	155	2,244	930	137	1,619	5,084	2,906	375
	Barisal^c^	SC / SW	1,699	0	0	0	11,905	0	0	0
	Bhola	SC	1,244	25	0	1,799	3,402	120	0	2,570
	Jhalokati	SC	535	0	0	0	4,708	0	0	0
	Patuakhali	SC	2,101	4,025	1,163	4,712	10,696	8,916	3,043	7,728
	Pirojpur	SC / SW	484	63	51	108	4,694	422	691	683

		*Sub-Total*	*6,218*	*6,357*	*2,144*	*6,756*	*37,025*	*14,541*	*6,640*	*11,357*

Chittagong	Chandpur^c^	SC	42	30	0	0	260	275	0	0

		*Sub-Total*	*42*	*30*	*0*	*0*	*260*	*275*	*0*	*0*

Dhaka	Faridpur^c^	SC / SW	2,128	0	0	0	7,321	0	0	0
	Gopalganj	SC / SW	528	78	0	0	3,338	333	0	0
	Madaripur^c^	SC / SW	723	0	0	0	4,192	0	0	0
	Manikganj^c^	SC	300	0	0	0	711	0	0	0
	Rajbari^c^	SC / SW	545	0	0	0	2,967	0	0	0
	Shariatpur^c^	SC	338	2	0	0	3,194	17	0	0

		Sub-Total	4,562	80	0	0	21,723	350	0	0

Khulna	Bagerhat	SW	132	54	0	1,362	979	284	0	3,418
	Chuadanga	SW	483	0	0	0		0	0	0
	Jessore	SW	501	159	0	118	4,192	1,189	0	420
	Jhenaidah	SW	539	0	0	0	6,478	0	0	0
	Khulna	SW	2	100	0	12,725	8	146	0	3,464
	Kushtia^c^	SW	594	0	0	0	4,329	0	0	0
	Magura	SW	651	0	0	0	3,770	0	0	0
	Meherpur^c^	SW	196	0	0	0	1,237	0	0	0
	Narail	SW	482	54	0	274	3,324	109	0	1,777
	Satkhira^c^	SW	1	31	0	2,418	13	369	0	1,974

		*Sub-Total*	*3,582*	*398*	*0*	*16,897*	*27,151*	*2,097*	*0*	*11,053*

		SC (Total)	8,711	6,361	2,144	6,730	47,939	14,688	6,640	11,206
		SW (Total)	5,693	504	0	16,923	38,220	2,574	0	11,204

		Grand Total	14,403	6,866	2,144	23,653	86,159	17,262	6,640	22,409

a Note that data include a 15 m exclusion buffer area adjacent to all waterways and water bodies for conservation purposes.

b Hydrological zone. SW = South-west. SC = South-central.

c Districts that are not entirely covered by the area of interest.

**Table 4:** Fallow and low intensity land intensification scenarios estimating the aggregate production of maize, wheat, and *boro* rice in observed surface water irrigation suitable areas, on medium-highland and higher landscape elevations for maize and wheat, and on medium-lowland and higher elevation for *boro* under high- (soil and surface water salinity each remain < 2 dS m^−1^), and medium- (soil and surface water salinity each remain between 2 and 4 dS m^−1^) suitability classifications^a^.

	Aggregate crop production (tons)								
	P = 0.25			P = 0.50			P = 0.75		
	Addressable land area^b^			Addressable land area^b^			Addressable land area^b^		
	25% area	50% area	75% area	25% area	50% area	75% area	25% area	50% area	75% area
Fallow land									
High potential									
Maize	26,125	52,251	78,376	20,918	41,837	62,755	18,315	36,630	54,945
Wheat	11,171	22,341	33,512	10,381	20,761	31,142	9,415	18,830	28,245
* Boro rice*	23,081	46,163	69,244	21,353	42,706	64,059	19,841	39,681	59,522

Medium potential									
Maize	14,618	29,236	43,855	11,705	23,409	35,114	10,248	20,496	30,744
Wheat	6,250	12,501	18,751	5,808	11,617	17,425	5,268	10,536	15,804
* Boro rice*	10,522	21,043	31,565	9,251	18,503	27,754	8,290	16,581	24,871

Low intensity land									
High potential									
Maize	1,60,787	3,21,573	4,82,360	1,28,741	2,57,482	3,86,223	1,12,718	2,25,437	3,38,155
Wheat	68,749	1,37,498	2,06,246	63,886	1,27,772	1,91,658	57,943	1,15,886	1,73,828
* Boro rice*	1,38,070	2,76,139	4,14,209	1,27,731	2,55,461	3,83,192	1,18,684	2,37,368	3,56,052

Medium potential									
Maize	36,199	72,398	1,08,598	28,985	57,969	86,954	25,377	50,754	76,132
Wheat	15,478	30,956	46,434	14,383	28,766	43,150	13,045	26,090	39,135
Boro	26,454	52,909	79,363	23,261	46,522	69,783	20,844	41,688	62,533

Totals									
Maize	2,37,729	4,75,459	7,13,188	1,90,349	3,80,697	5,71,046	1,66,659	3,33,317	4,99,976
Wheat	1,01,648	2,03,296	3,04,944	94,458	1,88,916	2,83,375	85,671	1,71,341	2,57,012
* Boro rice*	1,98,127	3,96,254	5,94,381	1,81,596	3,63,192	5,44,788	1,67,659	3,35,318	5,02,977

a Observations derived from geographically dispersed observations of farmer-managed experiments and crop fields between 2011 and 2014 for maize (*n* = 321 high- and 229 medium-suitability observations, wheat (*n* = 416 high- and 110 medium-suitability observations) and *boro* rice (*n* = 374 high- and 179 medium-suitability observations).

b Total addressable land area that can be served by decentralized surface water irrigation (124,690 ha, Table 3).

## Footnotes

1 All spatial information layers from this analysis are available in an open-source and interactive decision support tool for targeting surface water irrigation in southern Bangladesh (Schulthess et al., 2016), accessible at http://202.53.173.179/cimmyt/
